# Association of *NOS* Gene Polymorphisms with Sepsis-Related Complications in Secondary Peritonitis

**DOI:** 10.3390/ijms262110306

**Published:** 2025-10-23

**Authors:** Milica Rasic, Nela Maksimovic, Milka Grk, Marija Dusanovic Pjevic, Petar Rasic, Milos Svircev, Tatjana Damnjanovic, Dijana Perovic, Ana Djuranovic Uklein, Natasa Stojanovski, Milica Pesic, Ivana Novakovic, Krstina Doklestic Vasiljev

**Affiliations:** 1 Institute of Human Genetics, Faculty of Medicine, University of Belgrade, 11000 Belgrade, Serbia; nela.maksimovic@med.bg.ac.rs (N.M.); marija.dusanovic-pjevic@med.bg.ac.rs (M.D.P.); tatjana.damnjanovic@med.bg.ac.rs (T.D.); dijana.perovic@med.bg.ac.rs (D.P.); ana.djuranovic@med.bg.ac.rs (A.D.U.); natasa.stojanovski@med.bg.ac.rs (N.S.); milica.pesic@med.bg.ac.rs (M.P.); ivana.novakovic@med.bg.ac.rs (I.N.); 2 Department of Abdominal Surgery, Mother and Child Health Care Institute of Serbia “Dr. Vukan Cupic”, 11000 Belgrade, Serbia; petar.rasic@imd.org.rs; 3 Faculty of Medicine, University of Belgrade, 11000 Belgrade, Serbia; krstinadoklestic@gmail.com; 4 Department of Internal Medicine, General Hospital Pancevo, 23000 Pancevo, Serbia; milossvircev92@gmail.com; 5 Clinic for Emergency Surgery, University Clinical Center of Serbia, 11000 Belgrade, Serbia

**Keywords:** secondary peritonitis, sepsis, *NOS* polymorphisms, *NOS3*, *NOS2*, haplotypes, MODS, MOF, ARDS

## Abstract

Secondary peritonitis (SP) remains a major clinical challenge due to its high complication rates and it often results in sepsis and multi-organ dysfunction. This study investigated the association between four nitric oxide synthase (NOS) single-nucleotide polymorphisms (SNPs)—*NOS3* c.-786T>C (rs2070744), *NOS3* c.894G>T (rs1799983), *NOS3* 27 bp variable number tandem repeat (VNTR) (rs61722009), and *NOS2* (rs2297518)—and sepsis-related complications in 202 patients with SP. Demographic and baseline clinical characteristics, Acute Physiology and Chronic Health Evaluation (APACHE) II scores, Mannheim Peritonitis Index, and complications (multiple organ dysfunction syndrome (MODS), multiple organ failure (MOF), acute respiratory distress syndrome (ARDS), and sepsis) were analyzed for associations with the *NOS* gene variants. Haplotype analysis was also performed. No SNP showed an association with in-hospital mortality. However, the *NOS3* c.-786T>C TT genotype was significantly associated with an increased risk of MOF (*p* = 0.008), and remained independently associated after multivariate adjustment (*p*_MOF_ = 0.006). The T4bG haplotype was significantly more frequent among patients with MODS (*p* = 0.026), MOF (*p* = 0.048), and sepsis (*p* = 0.018). These findings suggest that *NOS* gene variants, particularly *NOS3* c.-786T>C and the T4bG haplotype, may potentially serve as biomarkers for risk stratification in critically ill patients.

## 1. Introduction

Secondary peritonitis arises from direct contamination of the peritoneum, typically resulting from a disruption of the integrity of the gastrointestinal or urogenital tract. It may occur due to perforation of hollow viscera or other pathological processes, such as intestinal ischemia, or intraperitoneal hemorrhage following trauma [1,2]. Despite significant advancements in diagnostic and treatment modalities, secondary peritonitis continues to be a major challenge in critical care settings globally, primarily due to its potentially life-threatening complications, such as intra-abdominal sepsis, and the high mortality rates associated with it [1]. Secondary peritonitis remains a leading cause of sepsis in intensive care unit (ICU) patients, underscoring the need for continued research and improved management strategies [2,3,4]. The Third International Consensus defines sepsis as a life-threatening organ dysfunction due to a dysregulated host response to infection [5], explaining the underlying pathophysiology of this complex condition. The response to infection may significantly differ between individuals and is believed to be influenced by several factors [6]. Research suggests that genetic predisposition plays a role in explaining the differences among hosts in terms of the clinical and pathophysiological aspects of sepsis, including the severity of complications and potential outcomes [7].

One of the key molecular players in sepsis pathophysiology is nitric oxide (NO), a potent vasodilator involved in regulating vascular tone, immune responses, and microcirculation. During sepsis, endothelial dysfunction and vascular dilation can lead to impaired blood flow and inadequate tissue oxygenation, which may contribute to organ dysfunction and septic shock [8,9,10]. While NO is essential for maintaining vascular tone by dilating blood vessels and lowering blood pressure under physiological conditions, excessive NO production in sepsis can worsen hypotension and exacerbate shock [11]. Genetic variations in NO synthase (NOS) genes, particularly *NOS3* and *NOS2*, have been shown to influence the production and function of NO. Specific single-nucleotide polymorphisms (SNPs) in *NOS3*, such as c.-786T>C (rs2070744) and c.894G>T (p.Glu298Asp, rs1799983), affect endothelial NOS (eNOS) activity, which may exacerbate vascular dysfunction and worsen sepsis outcomes by impairing vascular tone and increasing the susceptibility to shock [8,9,12]. Similarly, *NOS2*, which encodes inducible NOS (iNOS), is upregulated during sepsis and contributes to excessive NO production. Specific polymorphisms in *NOS*2, such as c. 1823C>T (p.Ser608Leu, rs2297518)**,** may enhance *NOS2* gene expression, exacerbating endothelial dysfunction and contributing to critical septic complications such as hypotension and multiple organ failure (MOF) [12,13]. Identifying genetic variants, particularly SNPs, through genome-wide association studies (GWASs) could provide valuable insights for improved diagnosis, prediction of sepsis severity, and personalized treatment strategies [14,15,16]. The exploration of such biomarkers, alongside traditional inflammatory markers, holds promise for improving outcomes in patients with secondary peritonitis and sepsis [17,18].

In this study, we conducted a comprehensive analysis of four SNPs located within the *NOS* genes, *NOS3* (including c.-786T>C (rs2070744), a 27 bp variable number tandem repeat (VNTR) in intron 4 (rs61722009), and c.894G>T, (p.Glu298Asp, rs1799983)) and *NOS2* c.1823C>T (p.Ser608Leu, rs2297518), to investigate their potential association with sepsis onset and the development of severe complications and outcomes in patients with secondary peritonitis. The previously published findings on these *NOS* gene variants are summarized in Table 1.

## 2. Results

### 2.1. Demographic and Clinical Characteristics of the Study Group

A total of 202 patients with secondary peritonitis were enrolled in the study. The demographic and clinical characteristics of the study population are presented in Table 2. The cohort had a mean age of 56.83 ± 17.7 years, with a slight male predominance (55.0%). The average Acute Physiology and Chronic Health Evaluation (APACHE) II score on admission was 10.63 ± 7.23, indicating moderate illness severity. The Mannheim Peritonitis Index (MPI) averaged 20.30 ± 7.46, reflecting the extent and severity of peritonitis in the cohort. Appendiceal perforation was the most frequent source of peritonitis, which was observed in 33.51% of the patients, followed by small bowel perforation (24.74%). Regarding the type of peritonitis, purulent peritonitis was the most prevalent, occurring in 80.73% of cases. The baseline laboratory findings are summarized in Table 2. Elevated white blood cell and neutrophil counts and increased C-reactive protein levels were consistent with systemic inflammatory response. The renal function parameters and bilirubin levels showed considerable variability, reflecting the heterogeneity in disease severity among the patients.

### 2.2. Allele and Genotype Frequencies

The frequencies of the *NOS* gene polymorphisms are summarized in Table 3. For *NOS3* c.-786T>C (rs2070744), the TT genotype was observed in 45.0% of the patients, and the T allele frequency was 0.69. The 4b/4b genotype of the *NOS3* 27 bp VNTR (rs61722009) was the most common (67.3%), with a 4b allele frequency of 0.83. The GG genotype of *NOS3* c.894G>T (rs1799983) occurred in 52.0% of the patients, with a G allele frequency of 0.72. For *NOS2* rs2297518 (alleles G/A based on genomic DNA; c.1823C>T based on HGVS (Human Genome Variation Society) coding nomenclature), the GG genotype was found in 58.9% of the patients, and the G allele frequency was 0.76. The genotype frequencies for all the analyzed SNPs were consistent with Hardy–Weinberg equilibrium (HWE). Overall, the allele frequencies closely resembled those reported in European reference populations.

### 2.3. Associations of NOS Genotypes with In-Hospital Mortality and Severe Complications

The associations between the *NOS* genotypes and in-hospital mortality are shown in Table 4. None of the investigated SNPs demonstrated a statistically significant association with mortality.

The relationship between the *NOS* gene polymorphisms and severe clinical complications (ARDS, MODS, and MOF) is summarized in Table 5. No significant associations were observed between any of the analyzed SNPs and sepsis. Among the analyzed polymorphisms, only the *NOS3* c.-786T>C (rs2070744) and *NOS2* c.1823C>T (rs2297518) variants showed statistically significant associations with major complications. The individuals carrying the *NOS3* c.-786T>C TT genotype exhibited an increased risk of both MODS (*p* = 0.017, OR = 2.67, 95% CI = 1.17–6.07) and MOF (*p* = 0.008, OR = 3.18, 95% CI = 1.31–7.69) compared to the individuals with at least one C allele (TC or CC). After applying the False Discovery Rate (FDR) correction (q = 0.05), the association between NOS3 c.-786T>C and MOF remained statistically significant (*p* = 0.032), whereas the association with MODS lost significance but showed a trend toward an increased risk (*p* = 0.068). On the other hand, the individuals carrying the *NOS2* c.1823C>T GG genotype showed an increased risk of both ARDS (*p* = 0.046, OR = 2.59, 95% CI = 0.99–6.77) and MODS (*p* = 0.045, OR = 2.46, 95% CI = 0.99–6.07) compared to the individuals with at least one A allele (GA or AA). After applying the FDR correction for NOS2 c.1823C>T, neither ARDS (*p* = 0.503) nor MODS (*p* = 0.090) remained significant. Given these findings, both the *NOS3* c.-786T>C TT and *NOS2* c.1823C>T GG genotypes were further evaluated in a multivariate context to assess their independent contribution. After adjusting for sex, age, source of peritonitis, and APACHE II score in the logistic regression models, only the *NOS3* c.-786T>C TT genotype remained significantly associated with a higher likelihood of developing MOF (*p* = 0.006, OR = 4.86, 95% CI = 1.56–15.13) and MODS (*p* = 0.047, OR = 3.67, 95% CI = 1.02–13.19). In the analyses of MOF and MODS outcomes, increasing age (*p*_MODS_ = 0.009; *p*_MOF_ = 0.030) and higher APACHE II scores (*p*_MODS_ = 0.000; *p*_MOF_ = 0.002) were consistently identified as significant predictors of adverse outcomes. In contrast, sex and source of peritonitis were not found to be significantly associated. The association of the *NOS2* c.1823C>T GG genotype with an increased risk of developing ARDS or MODS did not reach statistical significance in the logistic regression models (*p*_ARDS_ = 0.211; *p*_MODS_ = 0.146).

### 2.4. Haplotype Analysis

**Figure 1:**
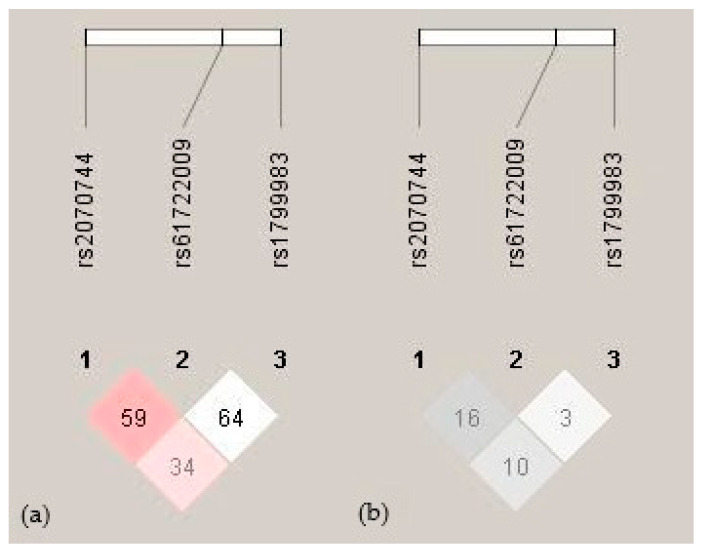
Linkage disequilibrium between *NOS3* gene polymorphisms. (**a**) Pairwise D′ values are indicated by red shading and numeric labels. Dark red indicates complete LD (D′ = 1) with an LOD > 2, while lighter shades represent lower values. (**b**) Pairwise r^2^ values are shown with gray shading and numeric values. Darker gray indicates a stronger correlation (higher r^2^) between variants.

Haplotype analysis was also performed to evaluate the combined effects of three *NOS3* polymorphisms (c.-786T>C (rs2070744), the 27 bp VNTR (rs61722009), and c.894G>T (rs1799983)) on clinical outcomes. The calculated linkage disequilibrium (LD) metrics (D′ and r^2^) are illustrated in Figure 1. No well-established haplotype block was identified within this cohort. Moreover, the frequencies of all the polymorphism haplotypes were consistent with the HWE. Analysis of the haplotype frequencies among the study participants revealed distinct haplotypes: T4bG, C4bT, T4bT, C4aG, C4bG, T4aG, and C4aT, with frequencies of 0.51, 0.13, 0.13, 0.11, 0.06, 0.05, and 0.02, respectively. Notably, the T4bG haplotype was significantly more frequent among the patients who developed severe complications. Specifically, its frequency was elevated in those with MODS (0.64 vs. 0.48; *p* = 0.026), MOF (0.61 vs. 0.48; *p* = 0.048), and sepsis (0.70 vs. 0.49; *p* = 0.018) compared to the patients without these complications. No other haplotypes demonstrated significant associations with these adverse clinical outcomes.

## 3. Discussion

This study provides new insights into how genetic variability in *NOS* genes affects clinical outcomes in patients with secondary peritonitis, a population at high risk for systemic inflammation and organ failure. Although no significant associations were found between the studied *NOS* variants and mortality, our findings revealed that the *NOS3* c.-786T>C (rs2070744) polymorphism, particularly the TT genotype, was linked to a significantly increased risk of MOF and showed a trend toward an increased risk of MODS. This association was significant for MOF and MODS after adjusting for potential confounders, suggesting that the *NOS3* c.-786T>C polymorphism independently contributes to severe organ failure in this population. The haplotype analysis further supported this finding, revealing that the T4bG haplotype of *NOS3* polymorphisms was significantly more frequent among the patients who developed MODS, MOF, and sepsis, indicating a potential combined effect of these variants on susceptibility to severe complications. Moreover, *NOS2* c.1823C>T GG genotype showed a significant association with a higher risk of both ARDS and MODS, although this association did not persist after multivariate adjustment.

The association between *NOS3* gene polymorphisms and sepsis has been the subject of several studies, which suggested a potential role of these variants in modulating susceptibility to and severity of the disease; however, the results remain inconclusive. Among these, the *NOS3* c.-786T>C promoter variant is known to affect *NOS3* gene expression, with the C allele typically linked to decreased transcriptional activity and, consequently, reduced NO production [19]. Interestingly, we found that the T allele, rather than the C allele, was associated with worse clinical outcomes, contradicting the expected protective role of higher NO bioavailability.

To our knowledge, our study is the first to assess the impact of selected variants on sepsis onset and the development of severe complications in patients with secondary peritonitis. Moreover, it is the first to report the T allele of *NOS3* c.-786T>C as a potential genetic risk factor for severe sepsis-related complications in this population. However, similar research has been conducted. Ma et al. found no correlation between the *NOS3* c.-786T>C variant and increased organ dysfunction or mortality in patients with severe sepsis [22], while Özkan et al. observed a higher frequency of the CC genotype and C allele in septic patients compared to healthy controls [10]. These discrepancies may reflect differences in study design, patient populations, or clinical outcomes, underscoring the complex role of *NOS3* variants in inflammation and critical illness. However, the association of the T allele with adverse outcomes in our cohort suggests a context-dependent effect. Some evidence showing that NO acts as a double-edged mediator in sepsis supports this interpretation, as both its deficiency and excess can contribute to organ dysfunction depending on the specific physiological context [11,26]. An animal study highlighting the importance of the *NOS3* gene demonstrated that *NOS3*-deficient mice experienced a greater degree of sepsis-associated MODS, with increased infiltration of mononuclear cells into tissues and heightened oxidative stress [27]. In secondary peritonitis, enhanced eNOS activity associated with the T allele may lead to excessive NO generation, promoting nitrosative stress through peroxynitrite (ONOO^−^) and other reactive nitrogen species (RNS), which can aggravate tissue injury and MOF [28,29,30,31]. This aligns with the studies underlying that modulation of the NO pathway should be finely balanced, as complete inhibition or uncontrolled upregulation of NO both worsen outcomes depending on when it occurs during the course of sepsis [26,32]. Consistently, clinical studies have shown that elevated circulating NO levels correlate with sepsis severity and adverse outcomes. Yu et al. reported that serum amyloid A and NO levels were positively correlated with APACHE II scores and mortality risk, reinforcing the idea that excessive production of NO contributes to disease progression [33]. Furthermore, recent insights summarized by Wu et al. highlight that the pathophysiology of sepsis involves a complex interplay of genetic predisposition, inflammatory signaling, and endothelial dysfunction, with eNOS-related pathways playing an important role [34].

In contrast to the significant associations observed for the *NOS3* c.-786T>C polymorphism, the 27 bp VNTR polymorphism in intron 4 (rs61722009) did not show a significant link with mortality or major complications in our cohort. Although the 4a allele has been previously associated with reduced plasma NO levels and decreased eNOS activity [20], as well as increased susceptibility to osteomyelitis [21], our study suggests that this variant has a limited impact on outcomes in secondary peritonitis. Regarding the *NOS3* c.894G>T (rs1799983) polymorphism, which causes a p.Glu298Asp substitution and affects eNOS protein stability and NO bioavailability [19], we did not observe a significant correlation with mortality or severe complications. This is consistent with the results of Özkan et al. [10] but contrasts with the findings of Ma et al. [22] and Martin et al. [9], who reported associations between the T allele and increased organ dysfunction or sepsis susceptibility. Additionally, Huttunen et al. [23] found that the T allele is linked to hypotension in *E. coli* bacteremia, suggesting that this variant may affect vascular function during infection.

Haplotype analysis provided additional insights into the combined effect of *NOS3* variants on clinical outcomes. In a study on healthy volunteers, Metzger et al. identified the C-4b-Glu haplotype (–786C, 4b, p.Glu298) as being associated with lower vascular NO production, thereby illustrating the functional relevance of specific variant combinations [35]. In contrast, Özkan et al. investigated haplotypes in septic patients and identified the C-4b-G haplotype (comprising-786C, 27 bp 4b, and 894G) as more frequent among those with sepsis, suggesting a role of this combination in increasing disease susceptibility [10]. Collectively, these findings underscore that *NOS3* haplotypes can modulate NO bioavailability and impact clinical outcomes in inflammatory conditions, though the specific combinations associated with an increased risk of adverse outcomes may differ across populations and disease contexts. Our results extend these observations to secondary peritonitis, emphasizing the potential contribution of specific *NOS3* haplotypes to organ dysfunction and severe complications. Further functional studies are warranted to elucidate how these combined polymorphisms affect eNOS expression and NO production in acute inflammatory states.

In the present study, the GG genotype of *NOS2* rs2297518 polymorphism (p.Ser608Leu), located in exon 16, initially showed a significant association with an increased risk of developing ARDS and MODS in patients with secondary peritonitis. In contrast, previous research identified the A allele as the one associated with an increased risk of sepsis and septic shock [12]. However, in our study, the association of the GG genotype of the rs2297518 polymorphism did not reach statistical significance in the multivariable logistic regression models, suggesting that the observed effect may be influenced by confounding factors such as sex, age, source of peritonitis, or APACHE II score. Unlike the other NOS isoforms, *NOS2* is not constitutively expressed and is upregulated in response to cytokines, bacterial lipopolysaccharides (LPSs), and other inflammatory mediators or pathogen-associated molecular patterns (PAMPs), leading to substantial NO production. This mechanism has been implicated in the pathophysiology of septic shock, particularly through profound vasodilatation and subsequent hypotension [30,36,37]. Previous studies have suggested a functional role for rs2297518 in sepsis. Wang et al. reported an increased risk of septic shock among carriers of the GA and AA genotypes in two independent Chinese cohorts [12] while Martin et al. identified a higher prevalence of another *NOS2* variant (rs374198) in septic patients compared to uninfected controls [9]. However, no direct correlation between genotype and circulating NOx levels was observed, promoting the idea that genotype alone may be insufficient to predict clinical outcomes.

Although *NOS2* polymorphisms have also been linked to chronic inflammatory diseases such as inflammatory bowel disease (IBD) [24,25], the role of rs2297518 may differ in acute settings. In very-early-onset IBD, iNOS overexpression contributes directly to chronic mucosal damage, whereas in acute conditions like peritonitis, its role may be more limited and influenced by multiple overlapping inflammatory pathways. Taken together, our data suggest that rs2297518 does not substantially influence clinical outcomes in secondary peritonitis, possibly due to the predominance of other clinical predictors such as age and APACHE II scores. Further studies across diverse populations and clinical settings are needed to clarify the broader relevance of *NOS2* in infectious disease susceptibility and progression.

A primary limitation of our study is the absence of functional validation of the identified genetic associations. While we discussed potential mechanisms involving NO production and nitrosative stress, they remain speculative without direct measurements such as NO levels or enzyme activity. Additionally, since this was a single-center study, the generalizability of our findings to other populations may be limited. Although the allele frequencies in our cohort align with European reference data, validation in diverse, multi-center cohorts is needed.

To advance our understanding of *NOS* gene variants in secondary peritonitis and sepsis, future research should integrate larger, multi-center cohorts; include functional analyses of variant-specific effects on NO production; and explore gene–environment and gene–pathogen interactions. Integrating genetic data with transcriptomic and proteomic profiling may help uncover the biological mechanisms linking *NOS* polymorphisms to clinical outcomes in acute inflammatory states.

In conclusion, genetic predisposition may influence the host inflammatory response, potentially offering a path toward risk stratification or targeted therapies in this critically ill population. Our findings suggest that *NOS3* gene variants, both individually and in haplotypic combinations, may potentially serve as biomarkers or therapeutic targets for secondary peritonitis. However, further functional studies and analyses across diverse populations are needed to translate these genetic insights into clinical applications.

## 4. Materials and Methods

### 4.1. Study Population

This cohort study was conducted at the Clinic for Emergency Surgery at the University Clinical Center of Serbia and the Institute of Human Genetics in Belgrade, Serbia. A total of 202 patients aged 18 years and older who were diagnosed with secondary peritonitis were enrolled after being treated at the Clinic for Emergency Surgery. We excluded patients in the terminal phase of malignant or chronic systemic diseases, those receiving steroid or immunosuppressive therapy, immunocompromised patients, and pregnant individuals to reduce potential confounding factors. The study was approved by the Ethics Committee of the Clinical Center of Serbia (No. 5030/2) and the Ethics Committee of the Faculty of Medicine, University of Belgrade (No. 1322/IX-71). The study included all patients who had undergone surgery due to peritonitis. Postoperative treatment followed standard protocols for abdominal infections, beginning with empirical broad-spectrum antibiotic therapy based on clinical suspicion of the cause of the infection. Antibiotic regimens were later adjusted according to antibiogram results obtained from microbiological analyses of intraoperatively collected peritoneal fluid, postoperative blood cultures, and swabs.

The APACHE II score was calculated for each patient using parameters collected within the first 24 h of admission to assess illness severity and the risk of complications or mortality [38]. Severe complications monitored included ARDS, MODS, MOF, and sepsis. The diagnosis of sepsis in patients treated before 2016 was based on the criteria established by the 2001 SCCM/ESICM/ACCP/ATS/SIS International Sepsis Definitions Conference [39]. For patients treated from 2016 onwards, the diagnosis followed the criteria outlined in The Third International Consensus Definitions for Sepsis and Septic Shock (Sepsis-3) [5]. Additionally, a specific MPI score was used to evaluate the prognosis of peritonitis for each patient [40]. Patient monitoring included clinical evaluations, laboratory tests (e.g., complete blood count, inflammatory markers, coagulation profile, and biochemical parameters), and imaging studies, including ultrasound, X-ray, and multi-slice computed tomography (MSCT). These procedures were carried out during hospitalization considering the length of stay in the ICU as well as the morbidity and mortality rates.

### 4.2. DNA Extraction and Genotyping

Molecular genetic testing was performed at the Institute of Human Genetics, Faculty of Medicine, University of Belgrade. Total genomic DNA was extracted from 5 mL of whole blood using the salting-out method [41]. The concentration and quality of the isolated DNA were assessed using a fluorimeter (Qubit 3.0, ThermoFisher Scientific, Waltham, MA, USA). Standard quality control procedures were applied for all SNP genotyping assays. To assess reproducibility, a randomly selected 10% subset of samples was re-genotyped in duplicate, resulting in a concordance rate exceeding 99%. The overall genotype call rate was 98.5%. Negative controls (no DNA template) were included in each PCR run to monitor for potential contamination. Additionally, samples with ambiguous genotype results were re-analyzed to ensure accurate genotype assignment.

#### 4.2.1. *NOS3* Genotyping

Genotypes for the *NOS3* 27 bp VNTR in intron 4 were identified using the polymerase chain reaction (PCR) method. Additionally, genotyping for the *NOS3* c.-786 T>C (rs2070744) and *NOS3* c.894G>T (rs1799983) SNPs was performed using PCR analysis followed by restriction fragment length polymorphism (RFLP) analysis. Table 6 provides detailed information on the primer sequences, reaction conditions, restriction enzymes utilized, fragment sizes, and corresponding genotypes.

#### 4.2.2. *NOS2* Genotyping

The *NOS2* c.1823C>T (p.Ser608Leu, rs2297518) SNP located in exon 16 was genotyped using the TaqMan SNP Genotyping Assay (assay ID: C_11889257_10, ThermoFisher Scientific, Waltham, MA, USA). The analysis was conducted in a 96-well plate using a 7500 Real-Time PCR System (Applied Biosystems, Foster City, CA, USA). The genotypes for rs2297518 are reported according to the genomic DNA strand, where G represents the reference allele and A represents the variant allele. The corresponding HGVS nomenclature is c.1823C>T based on the coding DNA sequence of *NOS2*. Each reaction was carried out in a final volume of 12.5 μL and included 2x Probe qPCR Mix EURx (EURx, Gdańsk, Poland), 1x specific TaqMan assay, and 5–50 ng of genomic DNA; the thermal cycling conditions recommended by the manufacturer were used.

### 4.3. Statistical Analysis

During the planning phase of the study, the minimum sample size was determined using G*Power software (version 3.1.9.7). Assuming an effect size of 0.3, a Type I error (α) rate of 0.05, and a statistical power of 0.95 with 2 degrees of freedom (df), the necessary sample size was 172 participants.

Genotype and allele frequency differences were analyzed using the Chi-square test or Fisher’s exact test depending on the sample size and assumptions about the expected frequencies. Deviations from HWE were tested using the simple Pearson’s χ^2^ test. Statistical analyses were conducted with SPSS software, version 16.0 (SPSS Inc., Chicago, IL, USA). For genetic association analyses, correction for multiple testing was implemented using the Benjamini–Hochberg step-up procedure for FDR with cut-off values q = 0.05. FDR correction was applied using the False Discovery Rate Online Calculator [42]. Haplotype analysis of the *NOS3* gene was performed using Haploview software 4.2 (Broad Institute, Cambridge, MA, USA) [43]. A *p* value of 0.05 or lower was considered statistically significant.

## Figures and Tables

**Table 1:** Overview of the *NOS* gene polymorphisms analyzed in this study and their functional relevance.

SNP (rsID)	Location/Type	Reported Functional Effect	Reported Clinical Associations
*NOS3* c.-786T>C (rs2070744)	Promoter region	The C allele reduces *NOS3* promoter activity and NO synthesis [19].	Hypertension, endothelial dysfunction, and sepsis [10,19].
*NOS3* 27 bp VNTR (rs61722009)	Intron 4	The 4a allele is associated with lower NOS3 expression and plasma NO levels [20].	Osteomyelitis, hypertension disorders, and obesity [19,21].
*NOS3* c.894G>T (rs1799983)	Exon 7, missense variant	The T allele causes eNOS protein instability and reduced NO bioavailability [19].	Sepsis, hypotension, and cardiovascular risk [9,22,23].
*NOS2* c.1823C>T (rs2297518)	Exon 16, missense variant	The T allele may increase iNOS activity and NO production [12].	Sepsis, septic shock, and inflammatory diseases [12,24,25]

Abbreviations: NOS—nitric oxide synthase; VNTR—variable number tandem repeat; eNOS—endothelial nitric oxide synthase; iNOS—inducible nitric oxide synthase.

**Table 2:** Demographic and baseline clinical characteristics of patients with secondary peritonitis.

Variable		Patients, n (%)/$\bar{x}$ ± SD
Total cases		202
Age, years		56.83 ± 17.7
Sex		
	Male	111 (55.0)
	Female	91 (45.0)
APACHE II score		10.63 ± 7.23
MPI		20.30 ± 7.46
Length of ICU stay, days		2.93 ± 9.35
Source of peritonitis, n (%)		
	Upper gastrointestinal perforation	42 (21.65)
	Small bowel perforation	48 (24.74)
	Large bowel perforation	21 (10.82)
	Appendiceal perforation	65 (33.51)
	Gynecological peritonitis	2 (1.03)
	Biliary peritonitis	16 (8.25)
Type of peritonitis, n (%)		
	Purulent	155 (80.73)
	Stercoral	23 (11.98)
	Biliary	14 (7.29)
WBC, 10^9^/L		14.2 ± 7.0
Neutrophils, %		84.30 ± 11.75
PLT, 10^9^/L		277.33 ± 105.17
Urea, mmol/L		8.55 ± 7.16
Creatinine, µmol/L		114.02 ± 83.80
Bilirubin, µmol/L		18.63 ± 23.75
C-reactive protein, mg/L		168.04 ± 150.71

Abbreviations: APACHE II Score—Acute Physiology and Chronic Health Evaluation II Score; MPI—Mannheim Peritonitis Index; ICU—Intensive Care Unit; WBC—White Blood Cell; PLT—Platelet.

**Table 3:** Frequencies of genotypes and alleles of the *NOS* gene SNPs in patients with secondary peritonitis.

SNP (rsID)	Genotype/Allele	Frequency (n, %)
*NOS3* c.-786T>C (rs2070744)	TT	91 (45.0)
	TC	95 (47.0)
	CC	16 (7.9)
	T	0.69
	C	0.31
*NOS3* 27 bp VNTR (rs61722009)	4b/4b	136 (67.3)
	4a/4b	62 (30.7)
	4a/4a	4 (2.0)
	4b	0.83
	4a	0.17
*NOS3* c.894G>T (rs1799983)	GG	105 (52.0)
	GT	80 (39.6)
	TT	17 (8.4)
	G	0.72
	T	0.28
*NOS2* c.1823C>T (rs2297518)	GG	119 (58.9)
	GA	68 (33.7)
	AA	15 (7.4)
	G	0.76
	A	0.24

Abbreviations: SNP—single-nucleotide polymorphism; *NOS*—nitric oxide synthase; VNTR—variable number tandem repeat. Note: Alleles are presented based on the genomic DNA strand. HGVS nomenclature is used for promoter and coding region variants.

**Table 4:** Association between *NOS* gene polymorphisms and mortality in patients with secondary peritonitis.

SNP	Genotype Group	Patients, n (%)	Survived, n (%)	Died, n (%)	*p*
*NOS3* c.-786T>C	TT	91 (45.0)	75 (82.4)	16 (17.6)	0.700
	TC + CC	111 (55.0)	101 (91.0)	10 (9.0)	
*NOS3* 27 bp VNTR	4b/4b	136 (67.3)	116 (85.3)	20 (14.7)	0.264
	4a/4b + 4a/4a	66 (32.7)	60 (90.9)	6 (9.1)	
*NOS3* c.894G>T	GG	105 (52.0)	90 (85.7)	15 (14.3)	0.532
	GT + TT	97 (48.0)	86 (88.7)	11 (11.3)	
*NOS2* c.1823C>T	GG	119 (58.9)	101 (84.9)	18 (15.1)	0.252
	GA + AA	83 (41.1)	75 (90.4)	8 (9.6)	

Abbreviations: SNP—single-nucleotide polymorphism; *NOS*—nitric oxide synthase; VNTR—variable number tandem repeat.

**Table 5:** Association between *NOS* gene polymorphisms and severe complications in patients with secondary peritonitis.

SNP	Genotype Group	ARDS, n (%)	*p*	MODS, n (%)	*p*	MOF, N (%)	*p*
*NOS3* c.-786T>C	TT	15 (16.5)	0.165	19 (20.9)	0.017 *	18 (19.8)	0.008 *
	TC + CC	11 (9.9)		10 (9.0)		8 (7.2)	
*NOS3* 27 bp VNTR	4b/4b	19 (14.0)	0.503	22 (16.2)	0.290	20 (14.7)	0.264
	4a/4b + 4a/4a	7 (10.6)		7 (10.6)		6 (9.1)	
*NOS3* c.894G>T	GG	17 (16.2)	0.143	17 (16.2)	0.439	16 (15.2)	0.296
	GT + TT	9 (9.3)		12 (12.4)		10 (10.3)	
*NOS2* c.1823C>T	GG	20 (16.8)	0.046 *	22 (18.5)	0.045 *	17 (14.3)	0.472
	GA + AA	6 (7.2)		7 (8.4)		9 (10.8)	

* Level of significance was 0.05, according to the Chi-square test or Fisher’s exact test. Abbreviations: SNP—single-nucleotide polymorphism; *NOS*—nitric oxide synthase; VNTR—variable number tandem repeat; ARDS—acute respiratory distress syndrome; MODS—multi-organ dysfunction syndrome; MOF—multiple organ failure.

**Table 6:** Polymerase chain reaction (PCR) and restriction fragment length polymorphism (RFLP) conditions.

Gene	SNP	Primer Sequences (5′-3′)	PCR Conditions	Product Size (bp)	Reaction Enzyme	Genotypes and Restriction Fragments (bp)
*NOS3*	c.-786T>C	F: CCCAGCAAGGATGTAGTGAC R: GTGTACCCCACCTGCATTCT	Denaturation: 94 °C for 1 min Annealing: 60 °C for 1 min Extension: 72 °C for 1 min	306	Pdil or NaeI (37 °C, 1 h)	TT: 306 CT: 306, 225, 81 CC: 225, 81
	27 bp VNTR	F: CTATGGTAGTGCCTTGGCTGGAGG R: ACC GCC CAG GGA ACT CCG CT		210/183	/	4b4b: 210 4b4a: 210, 183 4a4a: 183
	c.894G>T	F: AAGGCAGGAGACAGTGGATGG R: CCCAGTCAATCCCTTTGGTGC		248	MboI (37 °C, 5–15 min)	GG: 248 GT: 248, 158, 90 TT: 158, 90

Abbreviations: PCR—polymerase chain reaction; *NOS*—nitric oxide synthase; VNTR—variable number tandem repeat.

## Data Availability

The original contributions presented in this study are included in the article. Further inquiries can be directed to the corresponding author.
